# Families or Unrelated: The Evolving Debate in Genetic Association Studies

**Published:** 2012-05-29

**Authors:** David W. Fardo, Richard Charnigo, Michael P. Epstein

**Affiliations:** 1 Department of Biostatistics, University of Kentucky, 725 Rose St, Lexington KY 40536; 2 Department of Human Genetics, Emory University, 615 Michael St, Atlanta GA 30322

## Abstract

To help uncover the genetic determinants of complex disease, a scientist often designs an association study using either unrelated subjects or family members within pedigrees. But which of these two subject recruitment paradigms is preferable? This editorial addresses the debate over the relative merits of family- and population-based genetic association studies. We begin by briefly recounting the evolution of genetic epidemiology and the rich crossroads of statistics and genetics. We then detail the arguments for the two aforementioned paradigms in recent and current applications. Finally, we speculate on how the debate may progress with the emergence of next-generation sequencing technologies.

## Statistics and Genetics

Although statistical genetics is often considered a young field, its roots can be traced back as far as 1869 with Francis Galton’s “Hereditary Genius: An Inquiry into its Laws and Consequences” [1]. This work preceded his Nature article, “Typical Laws of Heredity,” which introduced the quincunx and formulated the empirical law of reversion (later regression) [2]. Other statistical pioneers contributed greatly to the field of genetics. Karl Pearson founded both *Biometrika* (1901) and the *Annals of Human Genetics* (1925), while R.A. Fisher, along with J.B.S Haldane and Sewall Wright, is credited with founding the field of population genetics. To succinctly epitomize the overlap between the fields of statistics and genetics, consider this quote from L.J. Savage: “Even today, I occasionally meet geneticists who ask me whether the great geneticist R.A. Fisher was also an important statistician” [3].

## Genetic Epidemiology

The process of genetic epidemiology has been summarized via the following stages: descriptive epidemiology, familial aggregation, segregation analysis, linkage analysis, fine mapping, genetic association, cloning, and characterization [4]. These stages are sometimes but not always conducted in linear order, and some stages are expanded. Historically, the progression of analytic thought proceeded sequentially from: (1) observations of phenotypic differences between populations, to (2) demonstration that disease runs in families, to (3) examination of feasible genetic susceptibility models, to (4) tracking the cosegregation of genetic markers and disease through families, to (5) narrowing the region of candidate genes, to (6) association analysis with candidate genes, to (7) cloning and mutation identification, and finally to (8) functional and structural characterization of a gene. In more recent years, analysts have been able to circumvent steps (3)–(6) for gene mapping by employing hypothesis-free genome-wide association studies (GWAS) that examine the association between a phenotype and 100K to > 1M single-nucleotide polymorphisms (SNPs) across the genome. Most commercial GWAS panels enable a near-comprehensive assessment of common trait-influencing variation across the genome.

The majority of classic analytic methods in genetic epidemiology, including segregation and linkage analyses, require pedigrees for study. However, in this editorial we focus on association studies (both candidate gene and GWAS strategies) where the subject recruitment paradigm is not automatically determined. Here, the question of which markers are correlated with a particular phenotype can be approached with unrelated individuals or families. Studies can analyze unrelated subjects (collected from population-based or case-control studies) using standard statistics from regression or categorical data analysis. In the case of families, a variety of study designs are possible and include the case-parent trio design, which collects and analyzes genotype data on both an affected proband and the proband’s parents using a statistic like the transmission disequilibrium test (TDT; [5]). The TDT has been generalized for use with broader pedigrees (such as those collected for linkage analysis) and a range of outcome types using statistics like the family-based association test (FBAT; [6,7]). The analytic strategies developed have their own inherent strengths and weaknesses which shape, in part, the choice of using unrelated subjects or families for association studies. And therein lies the debate.

## Candidate Gene and Genome-wide Association Studies

While not as contentious as the arguments between some statistical genetics pioneers (see e.g. [8] or [9]), the controversy on family- versus population-based genetic association studies is widely recognized. The 2009 annual meeting for the International Genetic Epidemiology Society featured a discussion session titled “Family studies: Are they still relevant? Pro vs. Con,” in which Dr. Nan Laird articulated the virtues of family studies while Dr. David Balding questioned their relevancy. The discussion was cordial, but the arguments on both sides were substantive.

Population-based association studies are generally regarded as more statistically powerful than family-based studies, and they are easier to implement and, thus, can recruit more subjects. However, the different canonical units for the two paradigms (i.e. a case-control pair versus a case-parent trio for association mapping of a complex disease) impede simple comparisons. The corresponding association metrics are on different scales, and the family trio requires 50% more genotyping than a case-control pair. Even so, most experts agree that a case-control study is more powerful than a trio study at a fixed cost [10]. One caveat to this in the context of GWAS is that family-based methods may exploit between-family information to screen promising markers before statistical testing [11,12]. These methods’ distinct approach to the multiple testing in examining hundreds of thousands of markers makes comparison with population-based strategies more complex. Metrics used for population-based association studies are most commonly standard statistics and can be implemented in nearly any statistical software. Family studies require more textured knowledge and specialized software. In addition, the analytic handling of missing parents can be difficult [13].

The bane of population-based association studies is the potential for confounding due to undetected population stratification, i.e., systematic differences in ancestral allele frequencies. Conversely, protection against such confounding is often furnished as a rationale for family-based studies. Much effort has been invested in correcting for population stratification in population-based studies, and this area of research remains active [14]. Although some consider this problem resolved for common genetic variants, rare variants still pose substantial problems [15, 16].

Arguments for recruiting families comprise two main themes: extra information provided by family members and robustness to population stratification. Minimizing genotyping error is a foremost goal within genetic studies, and microarray genotyping platforms have made this aspect of quality control (QC) particularly important. When association signals come at the tail of a distribution generated by hundreds of thousands of markers, as in GWAS, a small systematic bias can easily yield false positives. Families add resolution to detect Mendelian inconsistencies and filter subjects based on excess genotyping error not detected by standard QC methods [17]. Families also allow for markedly more accurate haplotype phasing [18] and the detection of parent-of-origin effects. Often families have been previously recruited for linkage studies, so in these cases the logistical difficulties are greatly assuaged. In sum, the choice between population-based and family-based paradigms amounts to balancing the cost savings and power gains of the former against the robustness and additional resolution of the latter.

## Next-Generation Sequencing Studies

The advent of next-generation sequencing has complicated the debate, as methods for exome- and whole-genome sequencing have introduced new criteria for comparing subject recruitment paradigms. Reliable detection of de novo mutations and rare variants using pedigrees [19] as well as the ability to verify that rare, pathogenic variants cosegregate within families support the usefulness of recruiting families. Whether this utility outweighs the extra costs and logistical burden is still up for debate.

The population- versus family-based debate is not as heated as that between Fisher and Wright [9], for example. Some have even circumvented the current debate by combining both paradigms (see [20] for references). Regardless, those on either side can readily agree that the debate will likely continue, as will the need for a broad range of innovative statistical methodologies. The evolving quest to discover and refine our knowledge of genetic modifiers and causes of disease susceptibility will rely on such innovations.
